# The role of lymphoid tissue SPARC in the pathogenesis and response to treatment of multiple myeloma

**DOI:** 10.3389/fonc.2022.1009993

**Published:** 2022-12-20

**Authors:** Nesreen Amer Ramadan Aly, Samia Rizk, Azza Aboul Enein, Nermeen El Desoukey, Hamdy Zawam, Manzoor Ahmed, Mohey Eldin El Shikh, Costantino Pitzalis

**Affiliations:** ^1^ Clinical and Chemical Pathology Department, Faculty of Medicine, Cairo University, Cairo, Egypt; ^2^ Centre for Experimental Medicine and Rheumatology, William Harvey Research Institute, Barts and The London School of Medicine and Dentistry, Queen Mary University of London, London, United Kingdom; ^3^ Clinical Oncology and Nuclear Radiation Department, Faculty of Medicine, Cairo University, Cairo, Egypt

**Keywords:** multiple myeloma, SPARC, bone marrow, follicular dendritic cells, germinal center reactions, tumor microenvironments, lymphoid neoplasia

## Abstract

**Background:**

Despite the significant progress in the treatment of multiple myeloma (MM), the disease remains untreatable and its cure is still an unmet clinical need. Neoplastic transformation in MM is initiated in the germinal centers (GCs) of secondary lymphoid tissue (SLT) where B cells experience extensive somatic hypermutation induced by follicular dendritic cells (FDCs) and T-cell signals.

**Objective:**

We reason that secreted protein acidic and rich in cysteine (SPARC), a common stromal motif expressed by FDCs at the origin (SLTs) and the destination (BM) of MM, plays a role in the pathogenesis of MM, and, here, we sought to investigate this role.

**Methods:**

There were 107 BM biopsies from 57 MM patients (taken at different time points) together with 13 control specimens assessed for SPARC gene and protein expression and compared with tonsillar tissues. In addition, regulation of myeloma-promoting genes by SPARC-secreting FDCs was assessed in *in vitro* GC reactions (GCRs).

**Results:**

SPARC gene expression was confirmed in both human primary (BM) and secondary (tonsils) lymphoid tissues, and the expression was significantly higher in the BM. Sparc was detectable in the BM and tonsillar lysates, co-localized with the FDC markers in both tissues, and stimulation of FDCs *in vitro* induced significantly higher levels of SPARC expression than unstimulated controls. In addition, SPARC inversely correlated with BM PC infiltration, ISS staging, and ECOG performance of the MM patients, and *in vitro* addition of FDCs to lymphocytes inhibited the expression of several oncogenes associated with malignant transformation of PCs.

**Conclusion:**

FDC-SPARC inhibits several myelomagenic gene expression and inversely correlates with PC infiltration and MM progression. Therapeutic induction of SPARC expression through combinations of the current MM drugs, repositioning of non-MM drugs, or novel drug discovery could pave the way to better control MM in clinically severe and drug-resistant patients.

## Introduction

Multiple myeloma (MM) is a post-germinal center (GC) neoplasm that originates in the secondary lymphoid tissues (SLTs) and accumulates in the bone marrow (BM) during disease evolution (1). Malignant transformation of plasma cells (PCs) is a multistep process involving genetic and molecular events associated with significant and irreversible alterations in the GC and BM microenvironments (2, 3). In SLTs, B-cell receptor (BCR) and CD40 signaling induces MYC-dependent proliferation of B cells in the GC dark zone (4) where they are at high risk of undergoing malignant transformation (5), being mutation prone during class-switch recombination (CSR) and somatic hypermutation (SHM) (6, 7). Follicular dendritic cells (FDCs) are critically involved in the activation-induced cytidine deaminase (AID)-dependent B-cell CSR and SHM, and as such, they crucially contribute to the development of MM founder PC clones within the GCs of SLT (8).

MM appears to progress almost universally from monoclonal gammopathy of undetermined significance (MGUS). The molecular basis for the initial transformation of normal PCs to the establishment of MGUS is unclear, but dysregulation of the family of cyclin D proteins (cyclins D1, D2, and D3) contributes to this transformation at the very early stages (9). Nevertheless, dysregulation of cyclin D expression is not sufficient in itself to drive the disease (10) since myelomagenesis involves sets rather than individual genes. For example, malignant transformation of B cells was inducible upon transduction with a combination, but not single genes, of IRF4, MYC, BMI1, and BCLXL resulting in the development of IgM-secreting MM‐like cells (11).

FDCs trap native antigens in the form of immune complexes (ICs) for extended periods of time which critically regulate the GC reactions (GCRs) (12–14). During the GCR, ICs, toll-like receptor (TLR) ligands, and collagen induce FDC activation (15–17), and activated FDCs promote CSR, SHM, and high-affinity immunoglobulin (Ig) secretion (7, 18–21). Retention of T-cell-dependent (TD) antigens on FDCs is essential for SHM and selection of high-affinity BCRs during Ig affinity maturation, and while this process is invaluable for generation of effective immune responses, it is prone to genomic errors that contribute to oncogenesis. In fact, almost all myelomas are initiated by mutations associated with TD responses and there is now substantial evidence that myeloma-initiating alterations are related to errors in CSR (7, 22).

Secreted protein acidic and rich in cysteine (SPARC, also known as osteonectin or BM-40) is a collagen-binding matricellular protein involved in the regulation of numerous cellular processes including immune cell networking and extracellular matrix (ECM) assembly (23–25). Actually, loss of SPARC expression in SLTs results in disruption of the ECM owing to defective collagen remodeling and disturbed cell–matrix interactions (26).

FDCs in SLTs secrete SPARC (27); however, the contribution of FDC-derived SPARC in myelomagenesis has not been fully explored. Here, we investigated SPARC expression in the BM of MM patients, human SLTs, and *in vitro* GCRs and showed that SPARC is inducible in FDCs by ICs and LPS and displays significantly higher levels in the MM BM compared with controls. Moreover, SPARC inversely correlated with BM PC infiltration, ISS staging, and ECOG performance of the MM patients, and supplementation of the lymphocyte cultures with FDCs inhibited several oncogenes associated with myelomagenesis *in vitro*.

## Materials and methods

### Patients and specimens

Ninety-four BM biopsies were obtained from 57 MM patients attending the Department of Clinical Oncology and Nuclear Medicine, Kasr Al Aini Faculty of Medicine, Cairo University (NEMROCK). Thirty-two men and 25 women, aged 29–75 years old, underwent biopsy between 2014 and 2018, and the samples were taken at the time of diagnosis and after treatment during follow-up assessments. Treatment protocols included (1) VCD: Bortezomib (Velcade)/cyclophosphamide/dexamethasone; (2) VAD: vincristine/doxorubicin (Adriamycin)/dexamethasone; (3) thalidomide or lenalidomide (either alone or added to previous regimens); and (4) Endoxan or melphalan/steroid regimens. After induction treatment for 3–4 months, patients were categorized into autologous stem cell transplantation (ASCT)-eligible and non-eligible, and “proceed to ASCT” was justified if the patient response to treatment ≥ partial response (PR). In addition, 13 BM specimens from control patients undergoing investigations for diseases other than MM, e.g., immune thrombocytopenic purpura or before splenectomy, were included. The control group consisted of four men and nine women aged 17–66 years, and they were assessed during 2017. The relevant patients’ medical records including clinical and laboratory parameters at the initial diagnosis and follow-up assessments were retrieved, analyzed, and correlated with SPARC expression.

The BM specimens were taken under local anesthesia from the posterior superior iliac spines and processed in the Bone Marrow Pathology Unit, Clinical Pathology Department, Kasr Al Aini Faculty of Medicine, Cairo University. The study was approved by the Research Ethics Committee (REC), Faculty of Medicine, Cairo University (Rec. No. N-76-2018). The samples were fixed in 10% neutral buffered formalin for 24 h, decalcified in EDTA solution for 24–48 h, and then embedded in paraffin (FFPE). The FFPE BM biopsies were shipped to Barts and the London School of Medicine and Dentistry, Queen Mary University of London, where the studies were conducted in William Harvey Research Institute (WHRI) and the Genome Centre. In addition, histological blocks of human tonsils and cryopreserved tonsillar single-cell suspensions were obtained from the Centre for Experimental Medicine and Rheumatology BioBank at WHRI and used in the studies.

PC infiltration of the BM was assessed according to the International Myeloma Working Group (IMWG) guidelines and the revised 4th edition of the WHO Classification of Haemato-lymphoid Tumors (28–30). Briefly, BM aspirates were stained with Leishman–Giemsa and examined at 10, 40, and 100× magnification. Approximately 200–500 cells were counted and myeloma PCs [ranging from near-normal (oval cells with round eccentric nuclei, abundant basophilic cytoplasm, and perinuclear hof) to pleomorphic forms] were identified, and the percentages were recorded. In addition, serial sections of BM biopsies were stained with H and E [and CD138 HRP for confirmation] and examined at 4, 10, and 40× and the PC percentages were assessed by at least three senior hematopathologists, and the agreed averages were documented.

### RNA extraction and qPCR

RNA was extracted from the paraffin-embedded blocks using the RNeasy FFPE Kit (QIAGEN, Catalog No. 73504) following the manufacturer’s instructions. Briefly, four 10-µm-thick FFPE sections per extraction were deparaffinized in a microcentrifuge tube followed by sample treatment with proteinase K and DNase and transfer to an RNeasy MinElute spin column. RNA was left to bind the RNA-binding membrane in the column, then the membrane was washed several times followed by RNA elution in RNase-free water. The concentration and purity of RNA were determined by measuring the RNA sample absorbance at 260 (A260) nm and 280 (A280) nm against RNase-free water as a blank. A NanoDrop spectrophotometer was used for the measurement, and a 260/280 ratio of ~2.0 was generally accepted for the purity of RNA. Samples were stored at -80°C until further processing.

Reverse transcription and quantitative PCR were performed to measure the expression levels of SPARC and other genes. Total RNA was reverse transcribed using a high-capacity cDNA reverse transcription kit (Applied Biosystems Cat. No. 4374966) according to the manufacturers’ instructions. A program of sequential 25°C/10 min, 37°C/120 min, 85°C/5 min, and 4°C/∞ cycles was used to generate cDNA, and the purity was evaluated using the NanoDrop spectrophotometer. The ratio of absorbance at 260 to 280 nm (260/280) was calculated, and a value of ~1.8 was generally accepted as pure DNA.

qPCR was carried out in Applied Biosystems™ QuantStudio™ 7 Flex Real-Time PCR System using TaqMan Gene Expression assays (**Table 1**) and Master Mix (Applied Biosystems Cat. No. 4369016). All reactions were run in 384-well plates using a 20-ml total reaction volume containing 100 ng cDNA, and each sample was run in triplicate. The 100 ng cDNA concentration was chosen based on optimization assays using 100, 200, and 300 ng concentrations. No template control and housekeeping internal controls were used with each assay. Amplifications were performed with cycle parameters set at 50°C for 2 min followed by denaturation at 95°C for 10 min then 40 cycles of denaturation at 95°C for 15 s and a combined annealing/extension step at 60°C for 1 min. Optimization experiments were carried out with four endogenous housekeeping controls (18S, GAPDH, ACTB, β2M), and 18S was found the optimal normalization gene for the assessment of FFPE BM tissue samples.

**Table 1:** TaqMan primers and probes used for qPCR performed in this study.

Gene	RefSeq	Gene expression assay ID
18S	GenBank mRNA: 18s_consensus.0	Hs03003631_g1
ACTB	NM_001101.3	Hs99999903_m1
BCL2	NM_000633.2	Hs04986394_s1
BMI1	NM_005180.8	Hs00409821_g1
CCND1	NM_053056.2	Hs00765553_m1
CCND2	NM_001759.3	Hs00153380_m1
CCND3	NM_001136017.3	Hs01017690_g1
FGFR3	NM_000142.4	Hs00179829_m1
GAPDH	NM_001256799.2	Hs02786624_g1
IRF4 (MUM1)	NM_001195286.1	Hs00180031_m1
MYC	NM_002467.4	Hs00153408_m1
PRDM1 (Blimp-1)	NM_001198.3	Hs01068508_m1
SDC1	NM_001006946.1	Hs00896424_g1
SPARC	NM_001309443.1	Hs00234160_m1
WHSC1/NSD2(MMSET)	NM_001042424.2	Hs00983720_m1
XBP1	NM_001079539.1	Hs00231936_m1
B2M	NM_004048.2	Hs00187842_m1

The average of amplification cycle threshold (C_T_) values for each sample was calculated where C_T_ is defined as the PCR cycle number at which the fluorescence signal crosses an arbitrary threshold. The target C_T_ values were normalized to the housekeeping gene, generating a delta CT value (ΔC_T_ = C_T_ gene of interest – C_T_ internal control) representing the amount of target amplicon relative to the internal control. The fold change in gene expression in two samples relative to the endogenous controls was calculated using the ΔΔC_T_ method where 2^-ΔΔCT^ = [(C_T_ gene of interest – C_T_ internal control) sample A – (C_T_ gene of interest – C_T_ internal control) sample B)].

### Protein extraction and Western blotting

Proteins were extracted from the paraffin blocks using the Qproteome FFPE Tissue Kit (Qiagen Cat. No. 37623) according to the manufacturer’s instructions. Briefly, three freshly cut tissue sections of 10–15 μm thickness each were deparaffinized in xylene and different grades of ethanol, then treated with β-mercaptoethanol-containing extraction buffer at 100°C for 20 min then at 80°C for 2 h with agitation at 750 rpm. The sample was then centrifuged for 15 min at 14,000 × g at 4°C, and the supernatant containing the extracted proteins was transferred to a collection tube, aliquoted, and stored at -20°C. The protein yield was quantified using RC DC Protein Assay Kit II (Bio-Rad, Cat. No. 500-0122). The protein concentrations were calculated by comparing the absorbance recorded for each sample against the absorbance recorded for a bovine serum albumin (BSA) standard curve.

The expression levels of SPARC proteins in the BM and tonsillar tissues were assessed. Protein lysates (15 µg) were enatured and separated using sodium dodecyl sulfate (SDS)-polyacrylamide gel electrophoresis (PAGE) under reducing conditions. Purified recombinant human SPARC protein (R&D, Cat. No. 941-SP) was used at an optimized dose of 5 ng, and a molecular weight marker was included. Samples were resolved on precast mini protein polyacrylamide gels for 60 min in a vertical electrophoresis apparatus at a constant 150 V current.

Proteins were subsequently transferred to a nitrocellulose membrane, then the membrane was blocked with 3% BSA for 1 h at room temperature. After blockade, the membranes were incubated at 4°C overnight with a primary antibody targeting SPARC (Thermo Fisher Scientific, Cat. No. PA5-78178) diluted in 3% BSA at 1:2,000. Consequently, the membrane was incubated with HRP-conjugated secondary antibody (Donkey Anti-Rabbit IgG; Thermo Fisher Scientific, Cat. No. A16029), diluted 1:10,000 in 3% BSA for 1 h at room temperature. Clarity Western ECL Substrates (Bio-Rad, Cat. No. 1705061) were used for detection, and membranes were exposed to Amersham films (GE Healthcare, Cat. No. 28906836) that were developed and fixed. Membrane stripping was performed using stripping buffer (Thermo Scientific, Cat. No. 21059), and the membrane was re-probed at room temperature for 1 h with peroxidase-conjugated secondary antibody against β-actin diluted in 3% BSA at 1:50,000 as a loading control. The blots were subsequently revisualized by the ECL detection system, and the bands’ densities were semiquantified and normalized to the loading control by blot densitometry using ImageJ software.

### Immunofluorescence and confocal imaging

Paraffin-embedded BM biopsies from control and MM patients were cut into 3 µm-thick sections and mounted on charged slides, then allowed to dry at room temperature. The sections were then deparaffinized in xylene and 100% ethanol, rehydrated, and processed for antigen retrieval using target retrieval solution pH 6 (Dako, code S1699). In addition, 10-µm cryosections were cut from OCT-embedded tonsillar tissues, air-dried, and fixed in ice-cold acetone for 15 min. The slides were rehydrated/blocked with 2% horse serum in PBS at room temperature for 15 min in a humidified chamber. Subsequently, sections were incubated at room temperature with primary Abs for 2 h followed by three times washing in PBS then incubation with secondary Abs for 1 h. Sections incubated with PBS instead of the primary antibody were used as negative controls. After incubation with the secondary Abs, the slides were washed in PBS, dried, and mounted with Antifade Mounting Medium (Vector Laboratories, Cat. No. H-1400) and examined with scanning confocal microscopy (Leica TCS SPE). Three lasers (488, 543, and 647 nm) were used, and image acquisition parameters were adjusted to 1,024 × 1,024-pixel density and 8-bit pixel depth. Emissions were recorded in three separate channels, and digital images were captured and processed with Leica Confocal Software LCS Lite. A quantitative analysis of fluorescence intensity was done using ImageJ to statistically compare fluorescence intensity for markers between different groups. The Abs used in this study are listed in (**Table 2**), and their concentrations were 10 µg/ml for FFPE sections and 5 µg/ml for frozen tissues.

**Table 2:** List of antibodies used in this study.

Primary Abs:													
Clone		Reactivity		Target		Host		Format		Vendor		Cat. no.	
Polyclonal		Human		SPARC		Rabbit		Unconjugated IgG		Thermo Fisher Scientific		PA5-78178	
MI15		Human		Syndecan-1/CD138		Mouse		Unconjugated IgG1		Dako		M7228	
CNA.42		Human		FDC		Mouse		Unconjugated IgM		Thermo Fisher Scientific		14-9968-82	
Y1/82A		Human		CD68		Mouse		Unconjugated IgG2		BioLegend		333802	
**Secondary Abs and other stains**													
**Clone**		**Reactivity**		**Target**		**Host**		**Format**		**Vendor**		**Cat. no.**	
Polyclonal		Mouse		IgM µ chain		Donkey		F (ab′)2—AF 488		Jackson ImmunoResearch		715-546-020	
Polyclonal		Mouse		IgG (H + L)		Donkey		F (ab′)2—AF 488		Jackson ImmunoResearch		715-546-150	
Polyclonal		Mouse		IgG (H + L)		Donkey		F (ab′)2—AF 647		Jackson ImmunoResearch		715-606-150	
Polyclonal		Rabbit		IgG (H + L)		Donkey		F (ab′)2—Cy3		Jackson ImmunoResearch		711-166-152	
Polyclonal		Mouse		IgG (H + L)		Donkey		F (ab′)2—Cy3		Jackson ImmunoResearch		715-166-150	
Not Applicable				DNA				TOTO-3 iodide		Thermo Fisher Scientific		T3604	

### Tonsillar FDC and lymphocyte enrichment

Single-cell suspensions were prepared by incubating tonsillar tissues in a cocktail of 8 mg/ml collagenase D (Roche, Cat. No. 11088858001) and 625 μg/ml DNase I (Sigma, Cat. No. 10104159001) for 45 min at 37°C in a humified 5%, then FDCs were isolated by positive selection using magnetic-activated cell sorting (MACS) separation as detailed in (12). The single-cell suspension was washed in RPMI 1640 (Thermo Fisher Scientific, Cat. No. 61870010) then incubated for 2 h at room temperature with purified mouse anti-human FDC monoclonal antibody CNA.42 (Thermo Fisher Scientific, Cat. No.14-9968-82) at 1 μg/10^6^ cell in MACS buffer. The cells were washed and centrifuged, and the cell pellet was resuspended in MACS buffer then incubated for 15 min at 2−8°C with 20 μl/10^7^ cells anti-mouse IgM microbeads. Subsequently, the cells were washed and the magnetically labeled FDCs were retained in MACS LS columns whereas the lymphocytes were collected in the flowthrough. FDCs were eluted from the MACS columns, and the enriched FDC and lymphocytes preparations were counted and assessed for viability using trypan blue exclusion.

### *In vitro* GCRs

Three different culture conditions, FDCs, lymphocytes, and FDC + lymphocytes, were set up in triplicates at 300 × 10^3^ cells/ml in RPMI 1640 (Thermo Fisher Scientific, Cat. No. 61870010) supplemented with 10% FBS (Thermo Fisher Scientific, Cat. No. 10500064) and 1% antibiotic–antimycotic (Thermo Fisher Scientific, Cat. No. 15240062). Each culture condition was either left untreated or treated with LPS (Sigma-Aldrich, Cat. No. L6143) (1 µg/ml) or aggregated human IgG (Sigma-Aldrich, Cat. No. I4506) (1 µg/ml) representing immune complexes. IgG complexes were prepared by dissolving 1 mg human serum IgG in 0.5 ml PBS and heating at 65°C for 30 min till the solution is noticeably opalescent.

Cultures were maintained for 5 days at 37°C and 5% CO_2_, then the cells were collected by centrifugation and the culture supernatants stored. RNA was extracted from the collected cells using RNeasy Mini Kit (QIAGEN, Catalog No. 74104), and its quantity and quality were measured. The expression of 15 selected genes involved in MM (**Table 1**) was quantified by qPCR, and the differences/changes in gene expression relative to the endogenous control were calculated using the comparative CT methods (2 ^-ΔCT^) and (2^-ΔΔCT^). The IgM and IgG levels in the culture supernatants were measured using IgM and IgG ELISA Kits (Bethyl Laboratories, Cat. Nos. E88-100 and E88-104). Enrichment of different GC cells, *in vitro* GC reconstitution, and stimulation with BCR-mediated and -non-mediated polyclonal activators have been detailed in earlier reports (20, 31–35).

### Statistical analysis

Comparisons between two groups were performed using the paired/unpaired Student T-test or Mann–Whitney test according to data normality. For every quantified measurement, the overall survival (OS) and progression-free survival (PFS) of MM patients were estimated using Kaplan–Meier estimators and the differences between groups of the same measurement were assessed with the log-rank test. Fisher’s exact test was used to compare proportions of a categorical outcome according to different independent groups. Spearman and Pearson correlations were used to estimate the correlation between numerical variables according to data normality. For ELISA assays, the Kruskal–Wallis test with Dunn’s multiple-comparison correction was used to compare the means of each sample with the means of every sample for each experimental condition. When comparing how a response was affected by two factors (the type of cell and type of treatment), a two-way ANOVA with Tukey’s posttest was used to determine whether there was a significant difference between the associated means. A *p*-value < 0.05 was considered significant. Data were analyzed and graphed using GraphPad Prism 7.

## Results

### Patients’ characteristics

**Figure 1:**
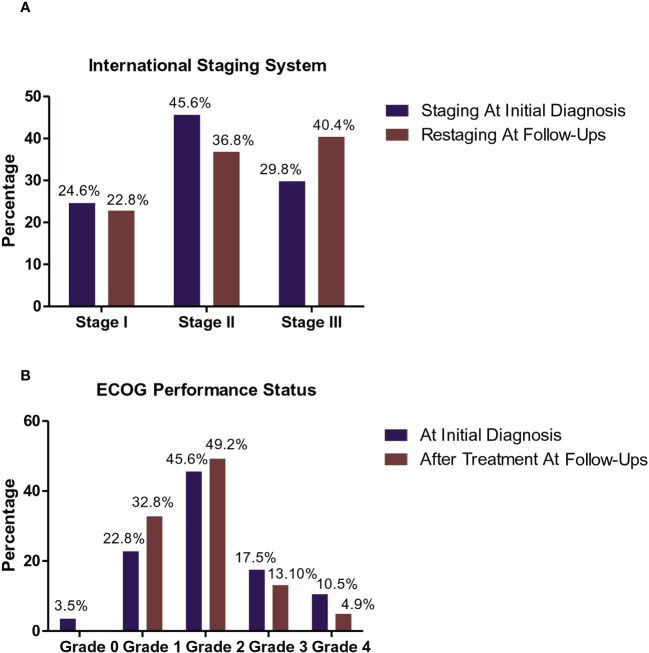
Staging and Eastern Cooperative Oncology Group (ECOG) performance status of the studied MM patients. International Staging System (ISS) Frequencies of MM patients at the initial diagnosis and following therapy **(A)**. At the initial diagnosis, 14/57 patients (24.6%) were classified as stage I, 26/57 (45.6%) with stage II, and 17/57 patients (29.8%) with stage III. While during follow-up after treatment, 13/57 patients (22.8%) were classified as stage I, 21/57 (36.8%) with ISS stage II, and 23/57 (40.4%) were of ISS stage III. ECOG performance status frequencies of MM patients at the initial diagnosis and during follow-up following therapy **(B)**. At the initial diagnosis, 2/57 (3.5%) of MM patients was recorded as grade 0, 13/57 (22.8%) of patients was recorded as grade 1, 26/57 (45.6%) of patients was recorded as grade 2, 10/57 (17.5%) of patients was recorded as grade 3, and 6/57 (10.5%) of patients was recorded as grade 4. While during their follow-up after treatment, 20/57 ( 32.8%) of MM patients was recorded as grade 1, 30/57 (49.2 %) of patients was recorded as grade 2, 8/57 (13.10 %) of patients recorded grade 3 performance status, and 3/57 (4.9 %) of patients recorded grade 4.

The clinical and descriptive data of the studied patients at the time of diagnosis are summarized in **Table 3**, and their staging and Eastern Cooperative Oncology Group (ECOG) performances before and after treatment are shown in **Figures 1A, B**, respectively.

**Table 3:** Descriptive and clinical characteristics of the MM patients at diagnosis.

Variables		Number	Percentage
Smoking	Yes	18	31.6 %
	No	39	68.4%
Associated comorbidities: e.g., diabetes mellitus, hypertension, cardiac diseases	Yes	16	28.1 %
	No	41	71.9 %
HCV	Positive	7	12.3%
	Negative	50	87.7%
HBV	Positive	2	3.5%
	Negative	55	96.5%
Complications: e.g., cord compression, neuropathy, paraparesis, paraplegia	Present	47	82.5%
	No	10	17.5%
Splenomegaly	Present	3	5.3%
	No	54	94.7%
Hepatomegaly	Present	12	21.1%
	No	45	78.9%
Lymphadenopathy	Present	5	8.8%
	No	52	91.2%
Bodily pain	Present	52	91.2%
	No	5	8.8%
Bone manifestations	Present	50	87.7%
	No	7	12.3%
Osteolytic bone lesions detected in radiological findings	Present	51	89.5%
	No	6	10.5%
Monoclonal protein	Present	31	54.4%
	No	26	45.6%
Hypercalcemia (Ca ^2+^ > 11 mg/dl)	Present	7	12.3%
	No	50	87.7%
Renal impairment	Present	12	21.1%
	No	45	78.9%
Anemia (Hb <10 g/dl)	Present	22	38.6%
	No	35	61.4%
Pathological fracture	Present	17	29.8%
	No	40	70.2%
Extramedullary disease	Present	12	21.1%
	No	45	78.9%
Coagulation profile	Abnormal	4	7%
	Normal	53	93%

Qualitative variables are presented as frequencies and percentages.

The peripheral blood and BM pictures before and after treatment of the MM patients are shown in **Table 4** where all parameters, apart from the platelet count, were significantly different before and after treatment. Furthermore, the laboratory profile of the patients (**Table 5**) showed significant differences in the serum Ca, total proteins, γ globulins, β globulins, α1 globulins, and the free light-chain kappa levels before and after treatment with *p* values of 0.04, 0.0079, 0.0195, 0.0303, 0.0156, and 0.0016, respectively. According to the European Society for Medical Oncology (ESMO) standards, four patients (7%) achieved complete response, two patients (3.5%) showed very good partial response (VGPR), 35 patients (61.4%) showed partial response (PR), three patients (5.3%) were categorized with less than partial response (<PR), and 13 patients (22.8%) were classified with progressive disease (PD). The OS of the patients ranged between 2 and 168 months with a mean value of 27.9 ± 26 months and a median of 22 months, whereas the PFS showed values between 0 and 129 months with a mean and median of 20.3 ± 19.9 15 months, respectively.

**Table 4:** Peripheral blood and bone marrow findings of the MM patients at initial diagnosis and during follow-up after treatment.

	Before treatment					After treatment					*p* value before/after treatment
	Mean	SD	Median	Min	Max	Mean	SD	Median	Min	Max	
TLC × 10^9^/L	7.33	3.66	6.6	0.8	21.9	5.65	2.35	5.10	1.9	12.8	0.0022
Hemoglobin gm/dL	10.5	2.57	11	3.9	16	11.9	2.13	11.8	6.7	16.8	0.0009
Platelets ×10^9^/L	245	96.2	243	8	544	239	103	239	7	534	
PC % in BM aspirates	19.3	17.3	14	2	80	10	15.1	5	1	90	<0.0001
PC % in BM biopsies	42.7	25.8	40	3	95	21	23.7	10	1	95	<0.0001

Quantitative variables are presented as mean ± standard deviation; p value < 0.05 is considered as statistically significant.

**Table 5:** Laboratory profiles of the MM patients at before and after treatment.

	Before treatment					After treatment				
	Mean	SD	Median	Minimum	Maximum	Mean	SD	Median	Minimum	Maximum
*Serum calcium total mg/dL	9.14	1.62	8.70	5.50	15	8.51	0.688	8.61	6.2	9.9
Serum albumin g/dL	3.19	0.723	3.3	1.40	4.50	3.29	0.618	3.4	1.3	4.2
Serum creatinine mg/dL	1.73	1.95	0.940	0.440	7.40	1.13	1.18	0.910	0.460	9.57
BUN mg/dL	24.3	24.3	16	6	128	15.3	8.85	14	7	71
β2 microglobulin mg/L	4.46	2.64	4.17	0.4	16	4.05	1.78	3.73	0.700	8.5
LDH U/L	258	154	219	83	911	209	56.3	207	120	481
*Serum total proteins gm/dL	8.43	1.99	7.9	5.3	16.7	7.59	0.830	7.5	6.2	9.9
SPEP albumin g/dL	3.39	0.501	3.31	2.28	4.3	3.5	0.414	3.5	2.47	4.48
SPEP albumin %	42.1	9.67	43.4	23.1	60.4	45.9	9.12	45.9	14.3	67.7
*SPEP gammaglobulins g/dL	2.5	1.94	1.98	0.15	10.5	1.68	1.16	1.55	0.200	5.16
*SPEP gammaglobulins %	26.5	14.7	22.9	2.3	62.9	20.4	10.4	20.9	3.07	50.7
*SPEP β globulins g/dL	1.12	0.321	1.11	0.5	2.05	1.25	0.338	1.22	0.650	2.70
*SPEP β globulins %	13.7	4.34	13.7	5.39	26.6	16.8	3.70	16.6	9.08	24.3
*SPEP α1 globulins g/dL	0.3	0.278	0.260	0.02	2.09	0.215	0.106	0.201	0.0290	0.650
SPEP α1 globulins %	3.54	2.51	2.89	0.370	16.1	2.92	1.25	2.81	0.710	7.85
SPEP α2 globulins g/dL	1.14	0.410	1.09	0.470	2.40	1.07	0.323	1.07	0.360	1.99
SPEP α2 globulins %	14.2	5.76	13.3	5.99	33.8	14.5	4.19	14.1	4.69	25.6
*Free light chain kappa mg/L	426	348	388	0.600	2290	320	133	336	1.5	674
Free light chain lambda mg/L	499	561	502	1.69	4330	461	224	507	2.6	1227
Free light chain ratio	89.7	96.7	87.7	0.009	747	78.9	42.5	86.2	1.10	216

SPEP, serum protein electrophoresis; BUN, blood urea nitrogen.

Quantitative variables are presented as mean ± standard deviation; p value < 0.05 is considered as statistically significant.

*Serum Ca, total proteins, γ globulins, β globulins, α1 globulins, and the free light chain kappa levels are statistically different before and after treatment with *p* values of 0.04, 0.0079, 0.0195, 0.0303, 0.0156, and 0.0016 respectively.

### SPARC expression and colocalization with FDCs in lymphoid tissues

**Figure 2:**
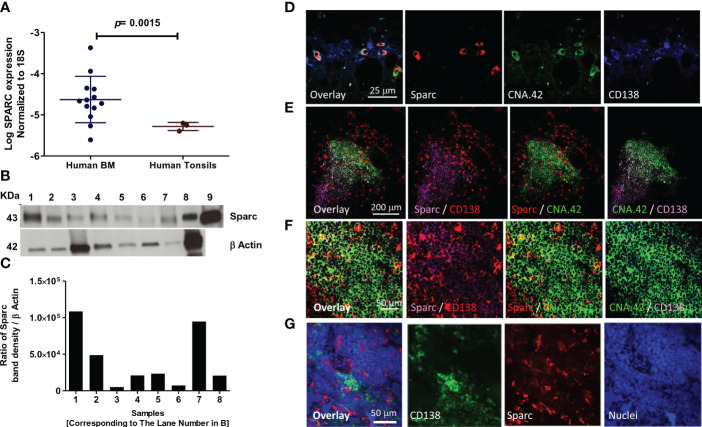
Expression of SPARC in normal human (primary and secondary) lymphoid tissues. Log SPARC expression level in normal human BM of 13 control patients (n = 13) and normal human tonsils (n = 3). **(A)** Western blotting of SPARC; β-actin was used as internal loading control, and an equal amount (15 μg) was loaded per lane. Lanes 1–7; BM tissue of seven control patients; 8, human tonsil; 9, human recombinant SPARC protein 5 ng (positive control) **(B)**. SPARC relative quantitation to β-actin using ImageJ; ratio of SPARC band density/β-actin band density **(C)**. Confocal imaging of SPARC (red), CNA42/FDCs (green), and CD138/PCs (purple) in normal human BM **(D)** and normal human tonsil **(E)**. Follicular GC light zone of tonsil at higher magnification **(F)**. The dual-color overlay of SPARC and FDCs (colocalization giving yellow/orange color; SPARC secretion from FDCs in the BM/tonsils), SPARC and CD138, and FDCs and CD138 (colocalization in GC light zone gives a white color) are shown. The triple-color overlay of SPARC, FDCs, and CD138 is shown, and the dark zone of the GC shows the PC differentiation. Confocal imaging of the SPARC (red), CD138 (green), and nuclear staining (blue) in normal human tonsil **(G)**. Sections are shown in three separate channels, and triple-color overlay of SPARC, CD138, and nuclei is shown.

SPARC expression at the RNA and protein levels was first assessed in the BM (primary lymphoid) and tonsillar (secondary lymphoid) tissues of control subjects before investigating its correlation with MM parameters and therapeutic response. Normalized to the internal control 18S, SPARC gene expression was significantly higher in the normal BM compared with the tonsils (**Figure 2A**). At the protein level, Sparc bands were detectable by specific mAb in Western blots of equally loaded BM and tonsillar lysates at a MW of ~43 kDa where recombinant Sparc positive control bands were seen (**Figure 2B**). Normalization of the Sparc band densities to β-actin (internal loading control) and analysis by ImageJ revealed protein expression in both tissues (**Figure 2C**) confirming the qPCR data.

We further investigated the cellular source of Sparc in the BM (**Figure 2D**) and tonsils (**Figure 2E**) and demonstrated by dual- and triple-color overlays of Sparc, CNA.42 [FDC-specific marker], and CD138 [PC marker] that Sparc co-localizes with FDCs in both tissues. High-magnification imaging of the GC light zone in tonsillar tissues revealed the intracellular and secreted forms of Sparc (**Figure 2F**). Furthermore, the triple-color overlay of Sparc, CD138, and nuclear staining (**Figure 2G**) validated the spatial proximity of Sparc and PCs in SLTs.

### SPARC expression in the BM of MM patients and the effect of treatment

**Figure 3:**
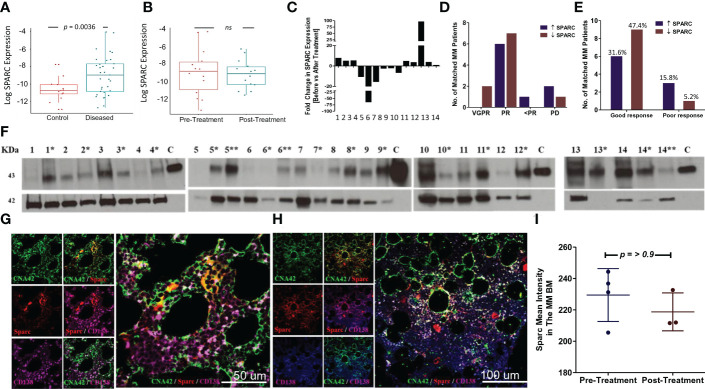
SPARC expression in the BM of MM patients and the effect of treatment. Log SPARC expression level in BM of diseased MM patients (before ttt) and normal BM of control patients **(A)**. The y-axis represents the relative expression of SPARC to 18S after log transformation, whereas the x-axis represents the patients’ status whether normal control patients or diseased MM patients before treatment. Unpaired T-test with Welch’s correction revealed a statistically significant difference between log SPARC expression (2^-ΔCT^) in MM diseased patients (n = 33) and the control patients (n = 13), p value = 0.0036. Log SPARC gene expression level (2^-ΔCT^) in 14 matched MM patients at baseline and post-baseline **(B)**. The y-axis represents the relative expression of SPARC to 18S after log transformation, whereas the x-axis represents the MM patients’ status at different time points (baseline and after treatment). Paired T test revealed no statistically significant difference between log SPARC expression (2^-ΔCT^) in matched MM patients before treatment (baseline) and after treatment (post baseline), n: number of patients (n = 14), p-value = 0.6364. SPARC gene expression (2^-ΔΔCT^) fold change (FC) after treatment in 14 matched MM patients **(C)**. SPARC FC (2^-ΔΔCT^) displayed inconsistent patterns, ranging from -64.7 to 96.75 reduced and upregulated folds due to treatment. SPARC gene expression FC stratification in 14 matched MM patients based on their response to therapy outcomes: VGPR/PR (good response) and <PR/PD (poor response) **(D, E)**. Fisher’s exact test did not show any statistically significant differences between the increased and decreased SPARC FCs as regards their response to therapy outcomes, p-value= 0.3034. Western blotting of BM tissue lysates show the changes in SPARC protein expression levels in 14 MM patients at their initial diagnosis (no asterisk) and following treatment [*, **; first and second follow-up respectively] **(F)**. β-Actin was used as internal loading control, and human recombinant SPARC protein 5 ng was used as a positive control, lane C. Confocal imaging of SPARC (red), CNA42/FDCs (green), and CD138/PCs (purple) in BM of a MM patient before treatment (BM infiltrated with 50% PCs) **(G)**, and after treatment (BM infiltrated with 20% PCs) **(H)**. Sections are shown in three separate channels. The dual-color overlay of SPARC and FDCs (colocalization giving yellow/orange color; shows SPARC secretion from FDCs in BM), SPARC and CD138, and FDCs and CD138 (colocalization giving a white color) are shown. The triple-color overlay of SPARC, FDCs, and CD138 is shown. Immunofluorescence semiquantitative analysis of SPARC fluorescence signal intensity in three MM patients before and after treatment using ImageJ **(I)**. Wilcoxon matched-pair test did not show any statistically significant differences in Sparc intensity, *p*-value = >0.9999.

Having validated the expression of SPARC in the BM, we sought to evaluate its expression levels in MM patients compared with controls. As shown in **Figure 3A**, BM Sparc expression was significantly higher in MM patients compared with control subjects; nevertheless, it was not significantly different before and after treatment of matched MM patients (**Figure 3B**). In matched MM patients, the fold change in SPARC gene expression before and after treatment [as calculated by the 2^-ΔΔCT^ equation] was variable. After treatment, the fold change of SPARC expression ranged from -64.7 [64.7-fold reduction] to 96.75 [96.75-fold increase] with a median of 1.006-fold reduction as shown in **Figure 3C**.

The change in SPARC gene expression after treatment was associated with different therapeutic outcomes in the MM patients. About 47.37% of patients had increased SPARC expression after treatment, 31.58% of whom showed good (VGPR/PR) and 15.79% showed poor (<PR/PD) response to therapy. On the other hand, 52.63% of the patients had decreased SPARC levels following treatment with 47.37% of this group displaying good (VGPR/PR) and 5.26% poor (<PR/PD) response to therapy. Overall, the fold change in SPARC gene expression was not significantly associated with a specific response to therapy in MM patients as illustrated in **Figures 3D, E**.

We further compared Sparc protein levels in the matched patients before and after treatment (**Figure 3F**), where the semiquantitative analysis of Western blots revealed that 73.3% of the patients showed low to no Sparc protein in the BM at the initial diagnosis then increased after treatment. Alternatively, 26.7% of the patients had shown high BM-Sparc before treatment, which was decreased after treatment. Most of the patients with increasing levels of Sparc protein under treatment showed good (VGPR/PR, 66.67%) response to therapy whereas 6.67% showed poor response (<PR/PD). On the other hand, 20% of patients with decreasing levels of Sparc upon treatment displayed good response to therapy (VGPR/PR) and the remaining 6.67% exhibited poor (<PR/PD) response.

Additionally, we assessed Sparc expression by immunofluorescence in the BM before (**Figure 3G**) and after (**Figure 3H**) treatment of MM. Confocal imaging revealed colocalization of Sparc with FDCs in the MM BM; however, the Sparc distribution and intensity quantified by ImageJ in three patients were not significantly different (**Figure 3I**).

Overall, the posttreatment changes in SPARC gene or protein expression were not significantly associated with a specific therapeutic response in MM patients.

### Expression of Sparc by macrophages *versus* FDCs in lymphoid tissues

**Figure 4:**
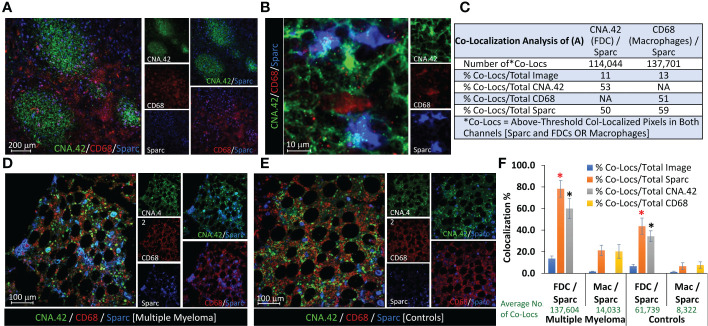
Contribution of FDCs and macrophages to Sparc expression in lymphoid tissues. Colocalization of FDCs (CNA.42/green), macrophages (CD68/red), and Sparc (blue) in **(A)** three adjacent tonsillar lymphoid follicles and the inter-follicular/peri-follicular regions. **(B)** High-magnification image of the tonsillar intrafollicular region showing colocalization of the same markers. **(C)** Colocalization analysis of **(A)** using the Colocalization Module of ImageJ as a representative of Sparc expression by FDCs and macrophages in SLTs (tonsils). BM biopsies from **(D)** an MM patient and **(E)** a control individual, showing Sparc colocalization with FDCs and macrophages. Single channel and dual overlays are also presented. Colocalization analysis of sections from at least three biopsies using the Colocalization Plugin of ImageJ is illustrated in **(F)** [Co-Locs = above-threshold co-localized pixels in both channels [Sparc and FDCs OR Macrophages]. The red star (*) signifies a *p* value of 0.032, whereas the black star (*) represents a value of 0.071 between the compared conditions. Error bars represent the standard error of means [SEM], and *p* < 0.05 is considered significant.

Sparc expression is not limited to FDCs, and other immune cells participate in its production in lymphoid and non-lymphoid tissues. Of major relevance to the B-cell follicles and GCRs, where the initial events of myelomagenesis occur, is macrophages that uniquely contribute to the removal of apoptotic B cells and released self-antigens during the GCRs [tingible body macrophages (32)], thus guarding against autoantigen recognition by the hypermutating BCRs and breach of self-tolerance. Macrophage expression of Sparc has been reported in hematological (36, 37) and solid cancers (38) and has been associated with enhanced cancer metastasis (39). Here, we sought to investigate the contribution of macrophages to Sparc expression in primary (BM) and secondary (tonsils) lymphoid tissues. As shown in **Figure 4**, Sparc colocalized with both the macrophage (CD68) and FDC (CNA.42) markers in the tonsils (**Figures 4A, B**) and BM (**Figures 4D, E**). In contrast to the tonsils where macrophage Sparc was marginally higher than the FDC Sparc (**Figure 4C**), BM Sparc in MM and control individuals was considerably colocalizing with FDCs (**Figure 4F**). The average number of colocalized pixels was higher with both cells in MM patients than in controls, and the percentage of FDC/Sparc-colocalized pixels to the total Sparc pixels was significantly higher (**Figure 4F** red star *p* = 0.032) in MM than in controls. Furthermore, despite the lower colocalization pixel numbers and percentages in controls than MM, the percentage of FDC/Sparc-colocalized pixels to the total FDC (CNA.42) pixels remained non-significantly unchanged (**Figure 4F**, black star *p* = 0.071) signifying the contribution of FDCs to BM Sparc expression in normal and diseased tissues.

### Correlation of BM SPARC expression with MM parameters and PC infiltration

**Figure 5:**
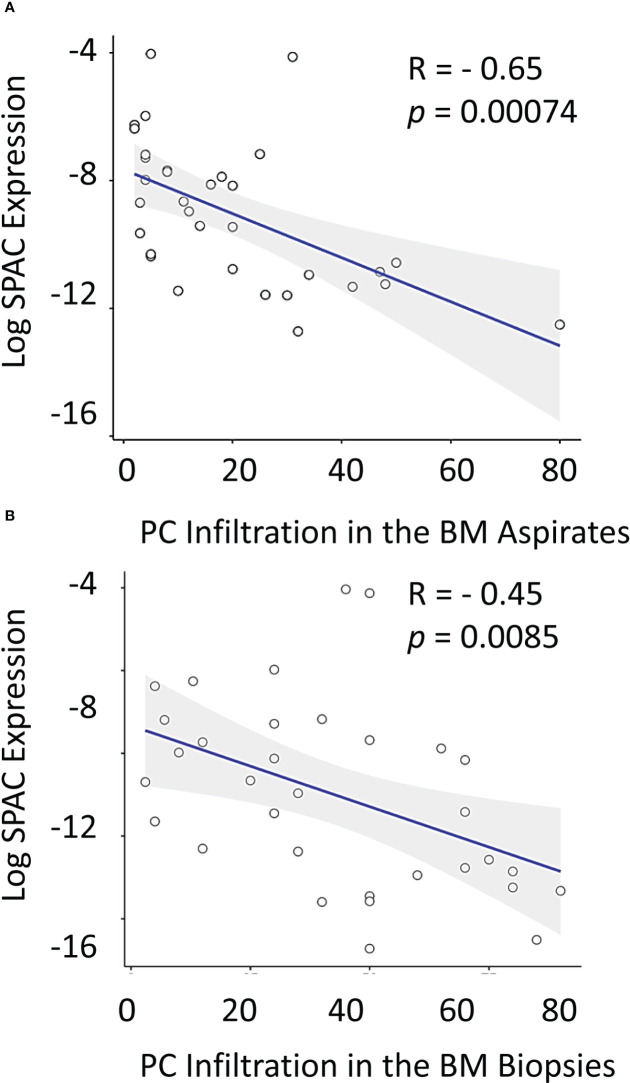
Correlation between log SPARC expression level and bone marrow plasma cell infiltration % in multiple myeloma patients at diagnosis. The y-axis represents the relative expression of SPARC to 18S after log transformation, whereas the x-axis represents the BM PC infiltration % in diseased MM patients’ (before treatment) in BM aspirates **(A)** and BM biopsies **(B)**. Pearson r correlation revealed a statistically significant inverse correlation between log SPARC expression levels and BM plasma cell infiltration % in MM patients (n = 33) at their initial diagnosis; BM aspirates with P-value= 0.00074, and r = -0.56, BM biopsies with *P*-value = 0.0085, and r = - 0.45. Linear regression is shown as a solid line.

**Figure 6:**
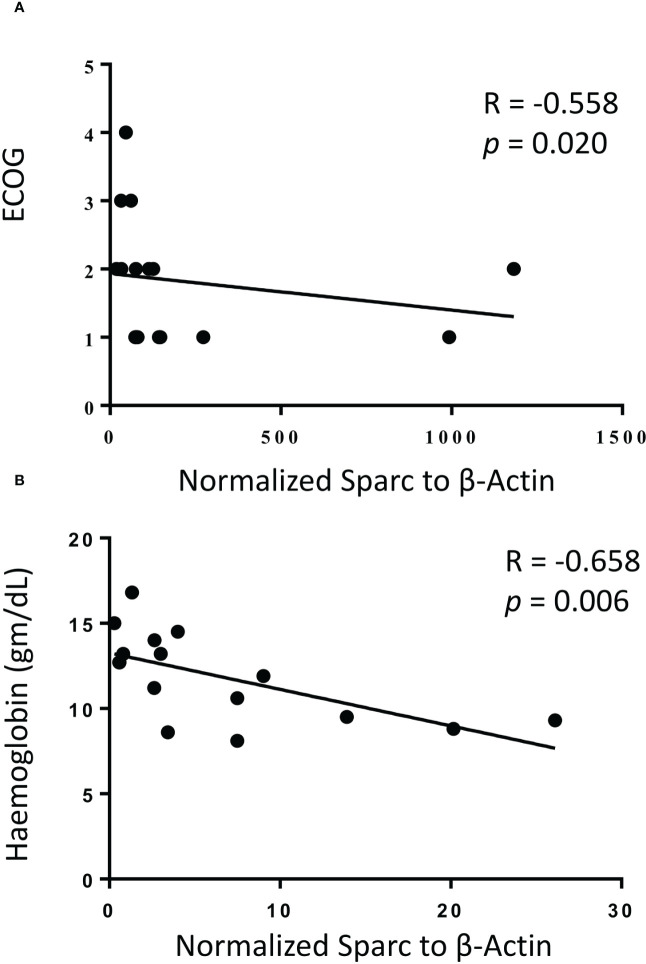
Correlation of bone marrow Sparc protein expression with ECOG performance status **(A)** and hemoglobin levels **(B)** following treatment in 14 matched multiple myeloma patients. Linear regression is shown as solid line, R = Spearman correlation, and *p* < 0.05 is considered significant.

While no significant correlation was noticeable between treatment and Sparc expression in the BM of MM patients, we reasoned that Sparc may be selectively correlated with specific disease parameters, and we sought to explore this. Significantly, our results showed that SPARC gene expression negatively correlated with PC infiltration in the BM aspirates and biopsies taken from MM patients before treatment (**Figure 5**). This inverse correlation was statistically significant with *p* values of 0.00074 and 0.0085 in the aspirates and the biopsies, respectively, and was associated, at the protein level, with a significant negative correlation with the patients’ ISS staging during posttreatment assessments (*p* = 0.028). Furthermore, the change in BM Sparc protein levels after treatment revealed a statistically significant negative correlation between Sparc and the patients’ ECOG (*p* = 0.020) and hemoglobin levels (*p* = 0.006) (**Figure 6**). The Sparc correlations with survival (**Supplementary Figure 1**) and several other clinical and laboratory characteristics were not statistically significant (**Supplementary Table 1**).

### Expression of SPARC and MM-associated oncogenes in *in vitro* GCRs

**Figure 7:**
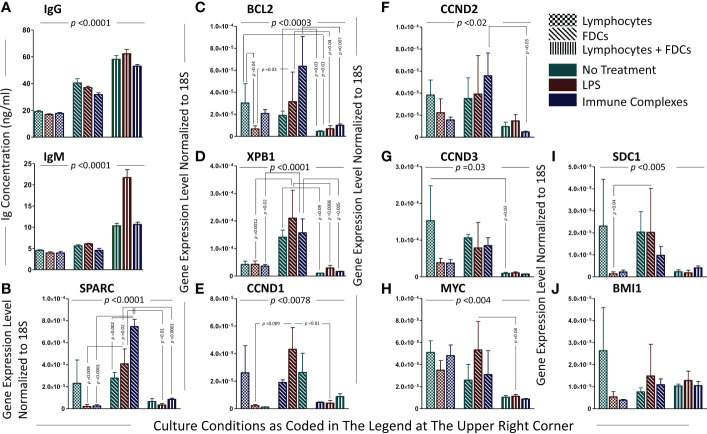
Analysis of plasma cell-associated genes and immunoglobulin secretion in the *in vitro* GCRs of SLTs. The levels of immunoglobulins secreted by PCs in the cell cultures of the *in vitro* GCRs among the different experimental conditions (evidence of B-cell activation and differentiation into PCs with antibody secretion); immunoglobulin IgG and IgM ELISA assays, respectively **(A)**. Data are presented as mean ± SEM, p-value <0.0001. Significant difference between the nine experimental conditions of the *in vitro* GCR cell cultures were detected using Kruskal–Wallis test with Dunn’s multiple-comparison correction in both assays. Each gene relatively expressed to 18S on lymphocytes, FDCs, and FDCs + lymphocytes in three experimental conditions (untreated, stimulated with LPS/ICs) **(B–J)**. Data are presented as mean ± SEM. Two-way ANOVA with Tukey’s posttest was used to compare the change in gene expression among the three types of cell culture under different treatments, and significant results were presented across the top of the graphs. Comparisons were then run between comparable conditions, and the significant differences were also shown.

The colocalization of Sparc with FDCs in tonsillar sections prompted our *in vitro* investigations to assess the effect of FDC activation on SPARC as well as 12 oncogenes involved in MM transformation. Tonsillar FDCs and lymphocytes (T + B cells at ~2:1 ratio) were isolated, and *in vitro* GCs were reconstituted with equal numbers of isolated cells in the presence or absence of FDC activators. FDC preparations from lymphoid tissues typically contain contaminating B cells (10%–20%) due to the strong FDC-B cell synapse resulting in B cells being trapped with the FDCs in the MACS columns during positive selection (12–14, 19). Consequently, the enriched FDC preparations show measurable levels of Ig secretion and B-cell-related genes’ expression. B-cell activation and plasma cell differentiation *in vitro* were confirmed by measurement of total human IgM and IgG in the culture supernatants. As illustrated in **Figure 7A**, IgM and IgG levels were significantly higher in FDC-supported than FDC-deficient cultures in the presence or absence of FDC activators (LPS or ICs). Moreover, FDC-enriched preparations produced higher levels of IgG than enriched lymphocytes alone, and LPS stimulation of FDC-lymphocyte cocultures induced higher IgM levels than IC treatment. We then evaluated SPARC and MM-related oncogenes’ expression in the different culture conditions as shown in **Figure 7B**. Normalized to the housekeeping gene 18S, SPARC was largely expressed by the enriched FDC preparations compared with lymphocytes or lymphocyte–FDC cocultures. Moreover, FDC stimulation with LPS or ICs induced significantly higher expression levels of FDC-SPARC. Similarly, BCL2 (**Figure 7C**) and XBP1 (**Figure 7D**) followed expression patterns comparable with SPARC in the different cellular conditions; however, the effect of LPS and IC treatments varied. In addition, CCND1, 2, and 3 (**Figures 7E–G**) were more expressed in FDC-enriched cultures compared with FDC–lymphocyte cocultures, and treatment of lymphocyte-only controls with LPS or ICs inhibited CCND1, 2, and 3 expression compared with untreated controls. Furthermore, MYC (**Figure 7H**) expression was higher in the lymphocytes’ controls compared with the FDC-enriched and FDC–lymphocyte cultures, and LPS stimulation inhibited MYC in the lymphocytes’ cultures compared with enriched FDCs. SDC1 (**Figure 7I**) expression was significantly higher in the enriched FDC preparations compared with other culture conditions. BMI1, IRF4, PRDM1, FGFR3, and NSD2 expression showed non-significant differences between the culture conditions; however, there was a trend of inhibition in lymphocyte cultures treated with LPS or ICs. BMI1 data shown in **Figure 7J**; IRF4, PRDM1, FGFR3, and NSD2 followed a relatively similar pattern.

### Effect of FDC coculture with lymphocytes on MM-associated oncogenes’ expression in *in vitro* GCRs

**Figure 8:**
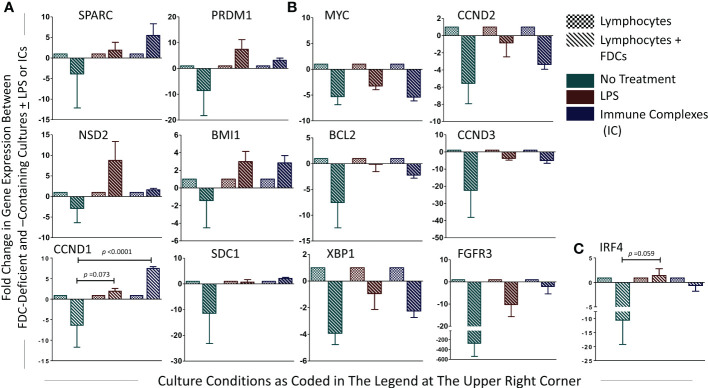
Fold change in PC-associated genes’ expression in lymphocytes + FDCs vs. lymphocytes. For each gene, fold change in the relative expression due to addition of FDCs to the lymphocytes was calculated using the equation (2^-ΔΔCT^), and the results were presented. Unpaired T-test was used to compare results in control (lymphocytes) with coculture groups (lymphocytes + FDCs) [whether untreated or treated with LPS or ICs], and significant *p* values were presented. Data represent mean ± SEM of three independent experiments. SPARC, NSD2, CCND1, PRDM1, BMI1, and SDC1 showed upregulated expression with LPS and IC treatment **(A)**, whereas MYC, BCL2, XBP1, CCND2, CCND3, and FGRF3 expressions were reduced **(B)**. IRF4 displayed increased and decreased expression with LPS and ICs respectively **(C)**, *p* value in LPS-stimulated cultures of IRF4 and CCND1 was 0.059 and 0.073, respectively.

Here, we directly investigated the effect of FDC coculture with lymphocytes on the expression of the MM oncogenes. The fold change in gene expression between lymphocytes and lymphocytes + FDCs under different treatment conditions untreated, LPS-treated, and IC-treated was calculated by the (2^-ΔΔCT^) equation, and the results are shown in **Figure 8**. Adding FDCs to the lymphocyte cultures without LPS stimulation or IC treatment tended to reduce oncogenes’ expression; however, this reduction did not reach statistical significance. Upon LPS and IC treatment, oncogenes’ expression showed two different trends. SPARC, NSD2, CCND1, PRDM1, BMI1, and SDC1 upregulated their expression (**Figure 8A**), whereas MYC, BCL2, XBP1, CCND2, CCND3, and FGRF3 expressions were reduced (**Figure 8B**). IRF4 displayed increased and decreased expressions with LPS and ICs, respectively (**Figure 8C**). Generally, the trends in oncogenes’ expression were not statistically significant apart from CCND1 where IC treatment significantly upregulated its expression as well as IRF4 and CCND1 in LPS-stimulated cultures where the *p* value was close to significance (0.059 and 0.073, respectively).

## Discussion

The lymphoid tissue microenvironment, including the distinctive stromal component, FDCs, is critically involved in MM initiation and progression. Herein, we investigated the association of SPARC, an FDC-secreted matricellular protein, with MM clinicopathological parameters and oncogene expression. We report that (1) Sparc colocalizes with FDCs in primary (BM) and secondary (tonsillar) lymphoid tissues of normal subjects and MM patients; (2) SPARC expression is significantly higher in the BM of MM patients compared with controls; (3) in MM patients, SPARC expression shows a significant negative correlation with PC infiltration of the BM before treatment; (4) posttreatment, approx. 47% and 53% of patients showed upregulation or downregulation of SPARC expression, respectively, with >50% of both groups displaying good response to therapy; and (5) in *in vitro* GCRs, a) SPARC was largely expressed by the enriched FDC preparations and FDC stimulation significantly upregulated higher expression levels of SPARC, b) in the absence of LPS or ICs, addition of FDCs to the lymphocyte cultures reduced oncogenes’ expression, and c) in the presence of FDCs, LPS or ICs induced SPARC, NSD2, CCND1, PRDM1, BMI1, and SDC1 and inhibited MYC, BCL2, XBP1, CCND2, CCND3, and FGRF3 gene expression.

SPARC expression by FDCs, as demonstrated in our *in vivo* and *in vitro* studies, is critically involved in promoting FDC structure and function and, consequently, the GCRs where normal and transformed PCs originate. In fact, the significant upregulation of FDC–SPARC expression upon stimulation by LPS and ICs could be vital to the modeling of the GC microenvironment, thus allowing effective FDC–B-cell interaction and PC differentiation. SPARC is required for collagen remodeling, and its loss results in disruption of the GC-ECM and impaired FDC network formation (40). We have previously reported that FDC stimulation by collagen type I induces dendrites and network formation (15), which partly explains disruption of the FDC networks when collagen maturation is defective in a SPARC-lacking GC microenvironment. Maintenance of well-established FDC networks is essential for proper GC assembly and lymphocyte trafficking during protective immune responses, and actually, SPARC expression in SLT is induced during the adaptive immunity to microbial infections (26, 41).

In the BM, our findings indicated that Sparc is expressed by CNA.42^+^ stromal cells and its expression was significantly higher in MM patients compared with controls. CNA.42 is a mAb that uniquely recognizes immature and mature human FDCs and is used for FDC labeling in cell lines and histopathological specimens (42–48). While our results displayed higher SPARC expression in the BM biopsies of MM, earlier reports showed both Sparc overexpression and downregulation in malignant PC compared with normal controls (49). SPARC expression and function is context and cell-type dependent (23, 50), and it is plausible that the effect of the BM microenvironment on SPARC expression, compared with isolated cells, could explain the difference in the results between biopsies, primary cells, and cell lines.

Our data indicate that both FDCs and macrophages produce Sparc in the B cell follicles, and both may have different and/or overlapping roles in health and disease. Compared with macrophages, FDCs are critically involved in B-cell activation, CSR, and SHM during the GC response (13, 19). FDCs provide several B-cell survival factors in the FDC–B-cell synapse including BAFF (34) and IL-6 (20). It is plausible that these factors together with FDC–Sparc act synergistically with other genetic and environmental factors in promoting myelomagenesis in MM patients. Whether macrophage–Sparc in primary and secondary lymphoid tissues contributes (positively or negatively) to myelomagenesis remains to be elucidated, and we plan to investigate this in future studies.

Importantly, SPARC expression inversely correlated with PC infiltration in the BM biopsies and aspirates from MM patients and this correlation was statistically significant. BM PC infiltration reflects higher disease burden (51) and worse outcome in MM, and our results obtained from the BM biopsies are in concordance with prior studies on the blood from MM patients reporting an overall trend to downregulation in the advanced stage of the disease (52). In fact, significant inverse correlations between the BM SPARC and the patient’s age, body mass index (BMI), ISS staging, and ECOG performance of the MM patients were also observed in our study supporting the association of low BM SPARC expression and increased MM risk, burden, and unfavorable prognosis. Shaped by the crosstalk with MM cells, the BM stroma is functionally different in MM from normal subjects (53), and it is conceivable that reduced FDC–SPARC expression is a manifestation of MM/stromal interaction resulting in FDC structural and functional inhibition. Actually, FDCs express receptors to several growth factors (54) and the expression of such receptors has been shown to be downregulated in advanced MM (55).

Almost 50% of MM patients investigated in this study showed upregulation of BM SPARC expression after treatment, suggestive of an impact of the treatment protocols on the BM microenvironment in MM. Indeed, bortezomib has been reported to upregulate SPARC expression in the BM stromal cells and this upregulation has an anti-adhesive effect on B cells in acute lymphoblastic leukemia (56). Similarly, lenalidomide (Revlimid) has been shown to induce >2-fold increase in SPARC expression in cultured cells isolated from patients with myelodysplastic syndromes, which may explain the anti-proliferative, anti-adhesion, and anti-angiogenic properties of this drug (57–59). Apparently, the current therapeutics of MM do not exert the same effect on SPARC expression in all patients, which may, in turn, be related to the heterogeneous nature of the disease and the individual response of the patients to treatment.

Predictably, FDC treatment with LPS or ICs induced significantly higher levels of SPARC in the FDC-enriched cultures, confirming our earlier studies that FDC-TLR4 and -FcγRIIB engagement with LPS and ICs respectively induces FDC stimulation and activated phenotype (16, 17, 54, 60). In fact, activated FDCs effectively co-stimulated B cells and induced PC differentiation and Ig secretion as illustrated by the significantly higher levels of Igs in the FDC–lymphocyte cocultures compared with lymphocytes.

Interestingly, in the absence of LPS or ICs, addition of FDCs to the lymphocyte cultures reduced the overall investigated oncogenes’ expression, implying the role of FDCs in driving normal PC differentiation. Whether this is directly or indirectly induced by FDC-SPARC and/or other FDC-derived B-cell co-stimulators remains to be verified. On the other hand, treatment of lymphocytes or FDC–lymphocyte cocultures with LPS or ICs resulted in inconsistent regulation of PC differentiation- and MM-associated genes (BCL2, XBP1, CCND1, 2, and 3, MYC, SDC1, BMI1, IRF4, PRDM1, FGFR3, and NSD2). In addition to the complex molecular networks implicated in the upregulation/downregulation of these MM-associated genes, this inconsistency could also be related to the effect of LPS and ICs on the heterogenous population of tonsillar human B cells (61). Furthermore, FcγRIIB engagement with ICs results in B-cell inhibition and plasma cell apoptosis (62–64), which could explain the significantly lower levels of Ig secretion in cultures treated with ICs compared with LPS.

It is noteworthy that PC differentiation and malignant transformation are multistep processes, and their gene expression profiles follow specific spatiotemporal cues. Certain genes mostly operate during the transition from B cells to PCs in SLTs; others show a dominant expression in the BM PCs, and several more display a significant expression in the BM MM cells (65). Our *in vitro* GCRs largely model human B-cell differentiation into PCs in the SLTs; however, the heterogenous nature of B cells in these cultures, the presence of cellular co-stimulators including T cells and FDCs, and the impact of LPS and ICs on human B cells partly explain the variable expression of the investigated oncogenes in our studies.

Remarkably, the enriched FDC fractions secreted comparatively higher levels of Igs than lymphocytes alone. In fact, the strong FDC–B-cell synapse makes it extremely difficult to prepare functionally active enriched FDC preparations without B-cell contamination, and in the presence of the FDC-derived secreted and membrane-bound co-signals [BAFF, APRIL, IL6, C3d, ICs] (12, 13, 19, 20, 66), B cells in the enriched FDC preparations survive and secrete Igs at levels even higher than those produced in FDC-lacking lymphocytes cultures. Additionally, compared with IgM, most of the secreted Igs in our *in vitro* GCRs are IgG owing to the prevalence (~80%) of class-switched long-lived memory B cells in the tonsillar preparations used in our cultures.

While retention of ICs and microbial TLR ligands activates FDCs and drive effective GCs, these FDC activators could also regulate genes contributing to PC differentiation and transformation. Indeed, hundreds of autoantibodies recognizing and forming ICs with tumor-associated antigens have been described (67) and almost 50% of MM patients are positive for anti-cardiolipin and lupus anti-coagulant autoantibodies (68–70). Moreover, the myeloma monoclonal (M) Ig itself has autoantibody activity against several tissue antigens including collagen IV, phospholipase A2 receptor, collagen VII, myelin-associated glycoprotein, gangliosides, and lipoproteins, and by forming ICs and activating complement, it induces extensive tissue damage and cytokine secretion (71). Furthermore, autoimmune patients with detectable autoantibodies are at a significantly increased risk of MGUS than autoantibody-negative patients (72, 73). Our results indicate that ICs in MM do not only induce tissue damage as previously reported but also regulate the ECM through induction of FDC–SPARC and modify the expression of several MM-associated oncogenes. Further pathophysiological roles and clinical significance of ICs and auto ICs in MM are planned to be investigated in follow-up studies.

Regarding TLRs, TLR4 is expressed on MM cell lines (74, 75) and is substantially high in the BM of MM patients (76–78). TLR4 activation in MM has a pro-oncogenic activity associated with increased proliferation, immune response evasion, protection against apoptosis, drug resistance, and poor prognosis (79–85). Our findings indicate that activation of FDC–TLR4 by LPS induced significantly high levels of SPARC, which inversely correlates with PC infiltration of the BM, ISS staging, and ECOG performance of the MM patients. Evidently, the pro-oncogenic activity of TLR4 on the MM cells is counteracted by FDC-SPARC upon TLR4 stimulation, and the outcome of this MM/stromal balance partly contributes to the severity and progression of the disease.

One of the limitations of our study is that we have not analyzed the effect of Sparc on the MM transcriptome. Studies using bulk and single-cell multi-omics of *ex vivo* sorted or *in vitro* induced MM cells as well as spatial transcriptomics/proteomics of the MM BM comparing Sparc+ *vs*. Sparc- territories are planned. Additionally, we plan to investigate the effect of different treatment regimens of MM in larger cohorts of patients on the serum and BM levels of SPARC and the correlation of these levels with disease progression and response to therapy.

In conclusion, we have shown Sparc expression by FDCs in the SLT, BM, and *in vitro* GCRs and that the expression was significantly higher in MM patients compared with controls. SPARC inversely correlated with BM PC infiltration, ISS staging, and ECOG performance of the MM patients, and *in vitro* addition of the SPARC-producing FDCs to lymphocytes globally inhibited the expression of several oncogenes associated with malignant transformation of PCs. Therapeutic induction of SPARC expression through combinations of the current MM drugs, repositioning of non-MM drugs, or novel drug discovery could pave the way to better control MM in severe and treatment-resistant cases.

## Data availability statement

The original contributions presented in the study are included in the article/**Supplementary Material**. Further inquiries can be directed to the corresponding author.

## Ethics statement

This study was approved by the Research Ethics Committee (REC), Faculty of Medicine, Cairo University (Rec. No. N-76-2018). The patients/participants provided their written informed consent to participate in this study.

## Author contributions

NA: study design, performed the experiments, data acquisition, data analysis and interpretation, figures, and manuscript preparation. ND, HZ: provided the experiments’ clinical data. MA: performed experiments and acquired data. SR, AA, ME, and CP: study design, interpretation of experimental results, manuscript writing and revision. All authors contributed to the article and approved the submitted version.
