# Supplementary material for: The role of lymphoid tissue SPARC in the pathogenesis and response to treatment of multiple myeloma
Source: Front Oncol. 2022 Dec 20;12:1009993. doi: 10.3389/fonc.2022.1009993 (PMC9807864; doi:10.3389/fonc.2022.1009993)
Supplement: Supplementary file 1 [file DataSheet_1.pdf]

Supplementary Materials

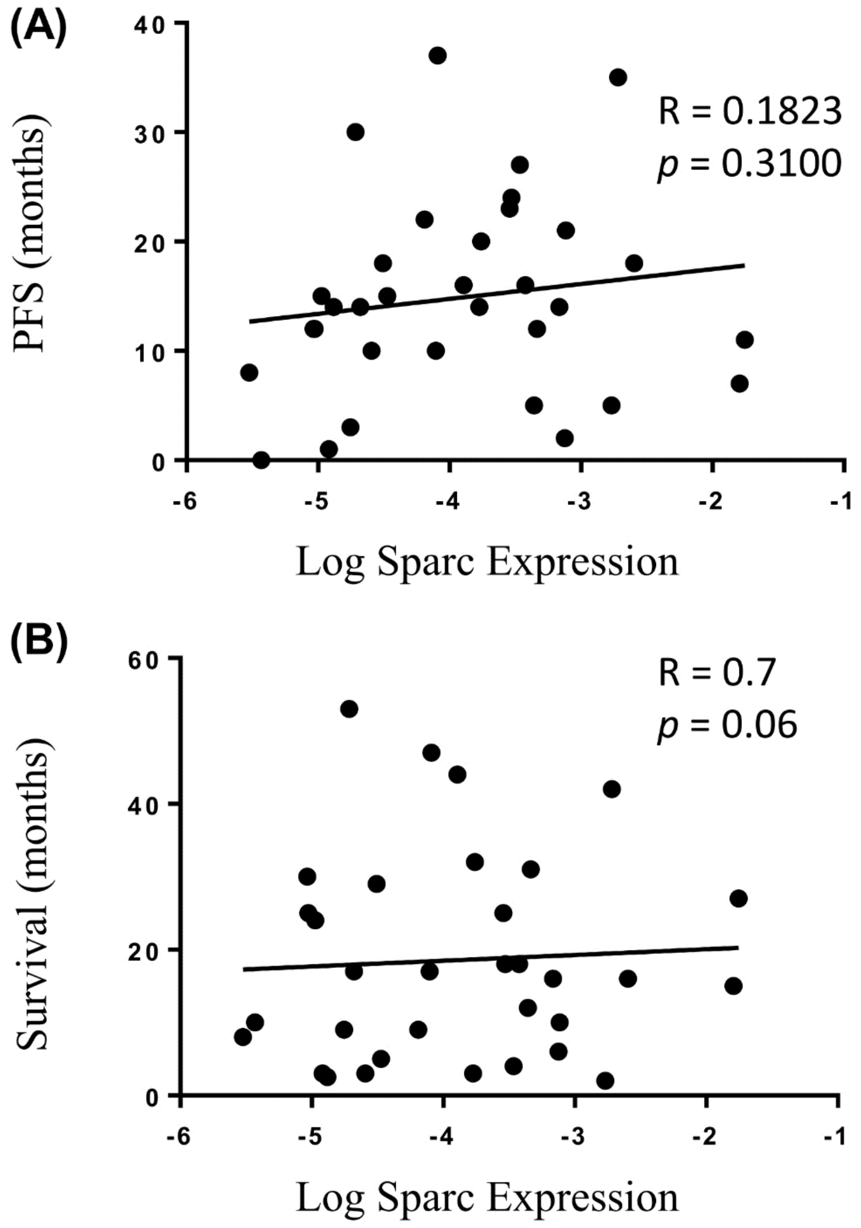

Supplementary Figure 1. Correlation of bone marrow log SPARC expression level with Progression Free Survival in months (PFS) (A), and Survival in months (B) in multiple myeloma patients at diagnosis. Linear regression is shown as solid line, R = Spearman correlation, and $p \leq 0.05$ is considered significant.

Supplementary Table 1: Correlation of SPARC relative gene expression ($2^{-\Delta CT}$) with the different disease parameters in MM patients at initial diagnosis and during follow up after treatment

**Clinical parameters**	**Before treatment**		**After treatment**	
	**r**	**p-value**	**r**	**p-value**
**Age (yrs)**	-0.1	0.56	-0.1197	0.3582
**Sex**	0.058	0.751	-0.090	0.492
**Smoking**	-0.086	0.634	-0.041	0.752
**Weight (Kg)**	-0.066	0.71	-0.09162	0.4825
**Height (cm)**	0.092	0.61	0.04344	0.7396
**BMI**	-0.1	0.58	-0.1195	0.3588
**ECOG**	-0.29	0.1	0.00043	0.997
**Associated comorbidities**	-0.111	0.537	-0.110	0.401
**HBV**	-0.149	0.409	-0.058	0.660
**HCV**	-0.195	0.277	-0.003	0.983
**Complications**	-0.049	0.788	0.245	0.057
**Splenomegaly**	-0.120	0.506	-0.286	**0.026***
**Hepatomegaly**	0.023	0.897	-0.113	0.386
**Lymphadenopathy**	-0.146	0.417	-0.113	0.387
**Bodily pain**	0.111	0.540	0.036	0.785
**Monoclonal protein**	-0.219	0.221	-0.061	0.638
**Pathological fracture**	0.155	0.388	-0.237	0.066
**Extramedullary disease**	-0.157	0.382	0.060	0.644
**Total Leucocytic Count (TLC)**	0.068	0.71	-0.2279	0.0773
**Hemoglobin (gm/dl)**	0.28	0.11	-0.1246	0.3388
**Platelets (Plts)**	0.42	**0.015***	0.08782	0.5009
**BMA Plasma cell infiltration%**	-0.557	**0.00074***	-0.05643	0.6658
**BMB Plasma cell infiltration%**	-0.45	**0.0085***	-2.664e-005	0.9998
**Renal impairment**	-0.357	**0.041***	-0.068	0.604
**Anemia (Hb < 10 g/dL)**	-0.345	**0.049***	0.015	0.908
**Bone manifestations**	0.053	0.768	0.039	0.765
**Osteolytic bone lesions**	0.010	0.957	0.041	0.756
**β2M**	-0.27	0.14	-0.02296	0.8606
**LDH**	-0.17	0.35	-0.006108	0.9627
**Serum Albumin**	0.18	0.32	0.2309	0.0734
**ISS**	-0.283	0.111	-0.186	0.152
**Serum Calcium Total**	-0.16	0.39	-0.127	0.3295
**BUN**	-0.27	0.12	-0.1471	0.2581
**Serum Creatinine**	-0.23	0.2	-0.09273	0.4772
**Coagulation profile**	0.244	0.172	0.066	0.613
**Serum Total proteins**	-0.0084	0.96	-0.01599	0.9026
**SPEP Albumin gm/dl**	0.15	0.39	0.263	**0.0406***
**SPEP Albumin %**	0.2	0.27	0.1858	0.1517
**SPEP Gammaglobulins gm/dl**	-0.19	0.3	-0.0211	0.8718

**SPEP Gammaglobulins %**	-0.17	0.33	-0.04543	0.7281
**SPEP β globulins gm/dl**	0.035	0.85	0.04458	0.7330
**SPEP β globulins %**	0.084	0.64	-0.02002	0.8783
**SPEP α1 globulins gm/dl**	0.044	0.81	-0.01971	0.8802
**SPEP α1 globulins %**	0.13	0.49	-0.04247	0.7452
**SPEP α2 globulins gm/dl**	-0.054	0.77	0.02004	0.8781
**SPEP α2 globulins %**	0.099	0.58	-0.006981	0.9574
**Free Light chain Kappa**	-0.3	0.089	-0.09421	0.4702
**Free Light chain Lambda**	-0.052	0.77	0.03517	0.7879
**Free Light chain ratio**	-0.17	0.33	-0.003887	0.9763
**Radiotherapy**	0.057	0.751	0.003	0.983
**BMT**	0.068	0.706	0.137	0.293
**Response to therapy**	0.431	**0.012***	-0.051	0.697
**PFS (months)**	0.1823	0.3100	-0.007056	0.9570
**Survival (months)**	0.0669	0.7114	-0.08214	0.5291

***= Statistically significant difference (P-Value<0.05)**
